# Weight-adjusted-waist index, inflammation, and cognitive performance in older adults: a cross-sectional analysis from the Hordaland Health Study

**DOI:** 10.3389/fragi.2026.1872693

**Published:** 2026-07-01

**Authors:** Ingrid Revheim, Lasse M. Giil, Ole Martin Steihaug, Hanne Rosendahl-Riise, Bård Erik Bogen, Ragnhild Eide Skogseth

**Affiliations:** 1 Department of Clinical Medicine, University of Bergen, Bergen, Norway; 2 Department of Geriatric Medicine, Haraldsplass Deaconess Hospital, Bergen, Norway; 3 Department of Clinical Science, University of Bergen, Bergen, Norway; 4 Department of Heart Disease, Haukeland University Hospital, Bergen, Norway; 5 Centre for Nutrition, Department of Clinical Medicine, University of Bergen, Bergen, Norway; 6 Department of Global Public Health and Primary Care, University of Bergen, Bergen, Norway

**Keywords:** abdominal obesity, ageing, cognitive performance, fat distribution, inflammation, weight-adjusted waist index

## Abstract

**Introduction:**

Obesity has been associated with reduced cognitive function, potentially through inflammatory and neuroinflammatory mechanisms. The weight-adjusted waist index (WWI) is a novel anthropometric measure capturing central adiposity. However, its association with cognitive performance has been scarcely studied, and it remains unclear whether inflammation influences this relationship. This study aims to examine the association between WWI and cognitive performance, and whether this association is explained by inflammatory markers.

**Methods:**

WWI was calculated as waist circumference divided by the square root of body weight (cm/√kg). Cognitive performance was assessed using a test battery comprising the Controlled Oral Word Association test (COWAT; verbal fluency), the Kendrick Object Learning Test (KOLT; memory), and a modified Digit Symbol Test (m-DST; processing speed). Inflammatory markers included C-reactive protein and the kynurenine-to-tryptophan ratio. Multivariable linear regression analyses were used to assess associations between WWI and cognitive test scores per standard deviation, and to evaluate potential attenuation by inflammatory markers.

**Results:**

A total of 2,066 community-dwelling older adults (55% women; median age 71 years [IQR 70–72]) from the Hordaland Health Study (1997–99) with complete data on WWI and cognitive tests were included in the cross-sectional analyses. Higher WWI was associated with lower cognitive performance across all tests: COWAT (ß −0.06 [95% CI −0.10, −0.02]), KOLT (ß −0.06 [95% CI −0.11, −0.02]), and m-DST (ß −0.09 [95% CI −0.12, −0.04]). Adjustment for inflammatory markers did not attenuate these associations. Body mass index, waist circumference, body fat percentage, and lean mass index were not significantly associated with cognitive performance.

**Conclusion:**

Higher WWI, reflecting greater central adiposity, was associated with lower performance across multiple cognitive domains. Inflammatory markers did not attenuate this relationship. Other anthropometric and body composition measures were not associated with cognitive performance. These findings suggest that central adiposity relative to body weight may be more relevant for cognitive health than overall body adiposity in older adults.

## 1 Introduction

Obesity has been linked to impaired cognitive function and increased risk of cognitive decline and dementia. Potential mechanisms include metabolic, inflammatory, and vascular pathways through which excess adiposity may adversely affect brain health, potentially promoting neurodegenerative disease and vascular pathology (Dye et al., 2017). However, the strength and consistency of these associations remain debated, and the relative importance of overall versus regional adiposity is not fully established.

Obesity, commonly assessed using body mass index (BMI), has been associated with both higher and lower risk of cognitive impairment (Wu et al., 2024; Anstey et al., 2011) While midlife obesity has been associated with increased risk of dementia, higher BMI in later life has been reported to show an inverse association with cognitive decline (Wu et al., 2024; Dahl et al., 2008) These discrepancies may partly reflect reverse causation due to weight loss in preclinical phases of dementia (Johnson et al., 2006), but they also highlight limitations of BMI as a proxy for adiposity.

BMI reflects overall body mass and does not differentiate between fat and lean mass nor account for fat distribution. Increasing evidence suggests that central adiposity, particularly visceral fat, is metabolically active and more strongly associated with adverse cardiometabolic and inflammatory profiles than generalized adiposity (Kolb, 2022; Saltiel and Olefsky, 2017) Failure to account for body fat distribution may therefore contribute to heterogeneity in studies examining obesity and cognitive outcomes.

The Weight-adjusted Waist Index (WWI) is a novel anthropometric measure reflecting central adiposity independent of overall body size (Park et al., 2018). WWI was developed by standardizing waist circumference (WC) to body weight, resulting in an adiposity index that is only weakly correlated with BMI (Park et al., 2018). This may address limitations of traditional anthropometric measures, including the “obesity paradox”, where higher BMI–and in some studies also higher WC–has been associated with lower morbidity and mortality risk in certain populations (Aggrawal et al., 2025; Clark et al., 2011).

By reducing the dependence on overall body size, WWI may better capture adverse fat distribution and muscle-fat imbalance compared with BMI and WC (Park et al., 2018; Zhou et al., 2024). Unlike BMI, which increases with both fat and lean mass, WWI has been shown to correlate positively with fat mass and negatively with muscle mass (Kim et al., 2022). Higher WWI has also been associated with increased risk of sarcopenia (Lee et al., 2026). This is particularly relevant in studies of cognitive health, as evidence suggests that altered body composition, including lower muscle mass and sarcopenic obesity, may be associated with cognitive decline (Vázquez-Lorente et al., 2025; Wu et al., 2025). WWI may therefore provide additional information on body composition beyond BMI or WC alone, making it a potentially relevant marker in studies of cognitive health.

Previous studies, primarily from US and Chinese cohorts, have reported associations between higher WWI and poorer cognitive performance (Li et al., 2024; Qiu et al., 2024), as well as accelerated cognitive decline (Qian et al., 2025). In contrast, higher BMI was associated with slower cognitive decline, whereas WC showed no significant association in the same study (Qian et al., 2025).

Obesity, particularly central adiposity, is closely linked to chronic low-grade inflammation (Saltiel and Olefsky, 2017; Hotamisligil, 2006; Gregor and Hotamisligil, 2011), which has been associated with cognitive decline and neurodegenerative processes (Walker et al., 2019). Beyond its systemic effects, obesity-related inflammation may also affect the central nervous system and promote neuroinflammatory processes (Miller and Spencer, 2014; Guillemot-Legris and Muccioli, 2017), which in turn has been linked to cognitive impairment and increased risk of neurodegenerative disease (Miller and Spencer, 2014; Zhang et al., 2023).

The essential amino acid tryptophan and its downstream metabolites, collectively referred to as kynurenines, have been associated with lower cognitive performance (Solvang et al., 2019) and dementia (Giil et al., 2017). Kynurenines are neuroactive compounds with immunomodulatory properties (Hwu et al., 2000). The kynurenines and the tryptophan-to-kynurenine ratio are closely linked to the innate immune system (Hwu et al., 2000) and serve as biomarkers of cellular immune activation. As WWI captures central obesity independent of overall body weight, it may reflect inflammatory and metabolic pathways particularly relevant to cognitive function.

We hypothesize that higher WWI is inversely associated with cognitive performance. Previous analyses from the same cohort reported associations between inflammatory biomarkers and cognitive performance (Solvang et al., 2019). Based on these findings, we hypothesize that inflammatory markers will attenuate the relationship between WWI and cognitive performance. We also hypothesize that body measures reflecting abdominal adiposity, such as WWI, WC, and waist-to-hip ratio (WHR), are more strongly associated with cognitive performance than measures reflecting overall body mass, such as BMI and body fat percentage (BF%).

Therefore, the aim of the present study was to investigate the association between central adiposity, measured as WWI, and cognitive performance in a large cohort of community-dwelling older adults, and to explore whether systemic inflammatory markers attenuate this association. A secondary aim was to explore the associations between other body measures, including BMI, WC, WHR, BF%, and lean mass index (LMI), and cognitive performance.

## 2 Materials and methods

### 2.1 Study population

The Hordaland Health Study (HUSK) was conducted in 1997–99. Participants born in 1925–27 were recruited into the Cognitive Sub-study. Details of the recruitment procedures have been described previously (Nurk et al., 2007). Of 2,841 individuals born in 1925–27, 2,197 (77.3%) agreed to cognitive testing. Cognitive testing, clinical measurements, and blood sampling were performed as part of the same examination. After excluding those without complete cognitive tests (n = 629) and those lacking data on WWI (n = 5), the final sample included in the current analyses comprised 2,066 participants.

### 2.2 Ethics

Participating subjects gave their written, informed consent prior data collection. The study was carried out in accordance with the Declaration of Helsinki, and the study protocol was approved by the Regional Committees for Medical and Health Research Ethics of Western Norway (REC number 2016/2208).

### 2.3 Assessment of cognitive function

Cognitive testing was performed at the study location by trained nurses. The cognitive test battery applied has been described in detail previously (Nurk et al., 2007) and included measures of memory, processing speed, and verbal fluency. Memory was assessed using the Kendrick Object Learning Test (KOLT) (Kendrick, 1985). Participants were shown slides containing objects and asked to recall as many objects as possible after each slide was removed. The procedure was repeated four times using a total of 70 objects, of which six appeared on all slides. One point was given for each correctly recalled object. Verbal fluency was assessed using an abridged version of the Controlled Oral Word Association test (COWAT) (Benton and Hamsher, 1989), where participants were asked to generate as many words as possible beginning with the letter S within 60 s. Performance on the COWAT reflects both executive functioning and language abilities (Shao et al., 2014). Processing speed and attention was assessed using a modified version of the Digit Symbol test (m-DST) (Smith, 1973), in which the number of correct digit-symbol matches completed within 30 s was recorded. Total test scores were used as continuous outcome variables in the regression analyses.

The original cognitive test battery also included a modified version of the Mini-Mental State Examination (m-MMSE) (Folstein et al., 1975), a short form of the Block Design (m-BD) (Wechsler, 1981), and the Trail-Making test Part A (TMT-A) (Reitan, 1958). Inspection of the score distributions revealed marked ceiling effects for both the m-MMSE and m-BD, limiting their ability to discriminate cognitive performance in this cohort. In addition, TMT-A scores showed an unexpected bimodal distribution suggestive of potential data recording or entry errors. As the validity of the TMT-A data could not be established with sufficient certainty, and because of the limited discriminatory capacity of the m-MMSE and m-BD, these tests were excluded from the analyses. Distributions of test scores are shown in Supplementary Figure S1.

### 2.4 Anthropometrics

The participants’ height (to the nearest cm) and weight (to the nearest 0.5 kg) were measured in light clothing without shoes. WC was measured to the nearest cm using a non-stretch measuring tape while the participants were standing in an upright position and breathing normally. BMI was calculated as the body mass in kilograms to the square of the height in meters. WWI was calculated by dividing the WC in cm by the square root of the body weight in kg (cm/√kg) (Park et al., 2018). WHR was calculated as WC in centimeters divided by hip circumference in centimeters. WWI, WC, BMI and WHR were included as continuous variables in the analyses.

### 2.5 Body composition

Body composition was assessed using dual-energy X-ray absorptiometry (DXA) with a stationary fan-beam densitometer (EXPERT-XL, software version 1.72–1.9; Lunar Corp., Madison, WI, United States) (Mazess et al., 1990). The coefficient of variation for fat mass was 1.9%. Total body fat mass and total lean body mass were obtained from the DXA scans. BF% was calculated as total fat mass (kg) divided by total body mass (kg) multiplied by 100. The LMI was calculated as total lean body mass (kg) divided by height squared (m^2^). BF% and LMI were included in the analyses as continuous variables.

### 2.6 Data collection

Self-administered questionnaires were used to collect data on current health status, medical conditions, physical activity, and sociodemographic characteristics including educational attainment. History of diabetes mellitus, stroke, and myocardial infarction was assessed by self-report and coded as binary variables (yes/no). Depressive symptoms were assessed using the depression subscale of the Hospital Anxiety and Depression Scale (HADS-D) (Zigmond and Snaith, 1983). A score of ≥11 indicated probable depression (yes/no) (Bjelland et al., 2002). Physical activity was assessed by self-reported average hours per week (h/week) of light- and vigorous-intensity activity during the previous year. Light and vigorous physical activity were categorized separately into four levels: none, <1 h/week, 1–2 h/week, and ≥3 h/week. Educational attainment was categorized into three levels based on highest completed education and total years of schooling: low (≤10 years, corresponding to primary education), medium (11–13 years, corresponding to secondary education), and high (≥14 years, corresponding to college or university education). Current smoking status was defined biochemically as plasma cotinine concentrations ≥85 nmol/L (Bevital, 2002).

### 2.7 Biochemical analyses

Non-fasting blood samples were collected in EDTA-tubes, centrifuged, and stored at −80 °C until analysis. Plasma concentrations of tryptophan (TRP), kynurenine (KYN), and cotinine were determined using liquid chromatography-tandem mass spectrometry under the conditions reported by Midttun et al. (2009). The ratio between KYN and TRP was calculated as KYN (µmol/L)/TRP (µmol/L) * 100.

Plasma high-sensitivity C-reactive protein (CRP) level was determined using an immune-MALDI (matrix-assisted laser desorption/ionization) mass spectrometry method (Meyer and Ueland, 2014). For CRP, the limit of detection was 0.2 ug/L, and within-day and between-day coefficients of variation were 5.5%–8.4% and 7.0%–11%–7%, respectively. All biochemical analyses were performed at Bevital AS (https://bevital.no).

### 2.8 Statistical analyses

#### 2.8.1 Descriptive statistics

Normality was assessed for all continuous variables by visual inspection of histograms and quantile-quantile (QQ) plots. Participant characteristics are described using summary statistics; continuous variables are expressed as means and standard deviations (SD), and categorical variables as counts and percentages. Highly skewed variables are presented as medians with the corresponding 25th and 75th percentiles (interquartile range).

#### 2.8.2 Regression analyses

Restricted cubic spline analyses (three to six knots) were applied to assess potential non-linear associations between anthropometric measures and each cognitive test. Non-linearity was evaluated by comparing linear and spline models using the Bayesian Information Criterion (BIC), with an improvement of ≥5 BIC points considered meaningful. Although formal tests indicated statistically significant non-linear terms for WWI with KOLT (*p* for non-linearity 0.015) and LMI with KOLT (*p* for non-linearity 0.001), spline models did not improve model fit according to the BIC (Supplementary Table S1). Thus, despite statistical evidence of minor deviations from linearity, the additional spline complexity did not meaningfully improve model performance. Visual inspection of spline plots did not reveal clinically meaningful deviations from linearity. Therefore, linear regression models were retained in all subsequent analyses based on interpretability and overall model fit.

Associations between z-standardized anthropometric measures (WWI, WC, WHC and BMI) and cognitive performance (z-standardized COWAT, KOLT, m-DST scores) were examined using multivariable regression models with stepwise adjustments for potential confounders selected *a priori* based on prior evidence linking anthropometric measures, body composition, and cognitive performance. Variables were z-standardized to facilitate comparison of effect sizes across cognitive domains. Model 1 was adjusted for age, sex, and educational attainment, established determinants of cognition (Hou et al., 2019; Gong et al., 2023; Ngandu et al., 2007). Model two additionally included lifestyle factors (physical activity and current smoking) (Carrasquilla et al., 2024; Fuentes et al., 2018; Campos et al., 2016; Mandolesi et al., 2018) and model 3 further adjusted for clinical conditions associated with both cognitive performance and body composition, including diabetes, myocardial infarction, stroke, and depression (Park et al., 2024; McCrimmon et al., 2012; Biringer et al., 2005; Pimenta et al., 2025; Liu et al., 2024; Shen et al., 2024). To explore whether systemic inflammation influenced the association between the primary exposure (WWI) and cognitive performance, inflammatory biomarkers (CRP and KTR) were included in a final model (Model 4). CRP and KTR were highly skewed on visual inspection of QQ plots and were therefore log-transformed to approximate normality before z-standardization. In supplementary analyses, inflammatory markers were entered individually to assess their independent contributions.

#### 2.8.3 Handling of missing data

Missing data for covariates were handled using multiple imputations (MI) under the assumption of missing at random. Imputations were performed using multiple imputation by chained equations, with all variables included in the analytical model also included in the imputation model. The exposure, outcome measures (KOLT, m-DST, and COWAT scores), as well as sex, age and CRP, had no missing values and were therefore not imputed. Categorical variables with missing values, including education (n = 166 missing), physical activity (n = 198 missing), diabetes (n = 30 missing), depression (n = 287 missing), stroke (n = 39 missing), myocardial infarction (n = 29 missing), and current smoking status (n = 12 missing), were imputed using logistic or ordinal regression models, as appropriate. Continuous variables including kynurenine (n = 32 missing) and tryptophan (n = 32 missing) were imputed using linear regression models. A total of 20 imputed datasets were generated to ensure adequate precision given the proportion of missing data. Estimates and standards errors were pooled according to Rubin’s rules.

#### 2.8.4 Subgroup analyses

Subgroup analyses were conducted among participants with available body composition data obtained by DXA. These analyses were performed to examine whether associations between body composition and cognitive performance were consistent with those observed for the primary exposure (WWI). As DXA measurements were available for 1,432 participants, the analyses were restricted to these subjects. Z-standardized body fat percentage and LMI were applied as the exposure variables in the analyses. Associations with cognitive performance (z-standardized COWAT, KOLT, and m-DST scores) were assessed using linear regression models. The same stepwise adjustment strategy as described above was applied, including Models 1–3. Missing data for covariates were handled using the MI model described above with 20 imputed datasets. Details can be found in supplements.

Statistical analyses were conducted in *Stata* for Windows, version 19.5 (StataCorp LLC, College Station, TX, United States). All tests were two-tailed with a significance level of 0.05.

## 3 Results

### 3.1 Participant characteristics

**FIGURE 1:**
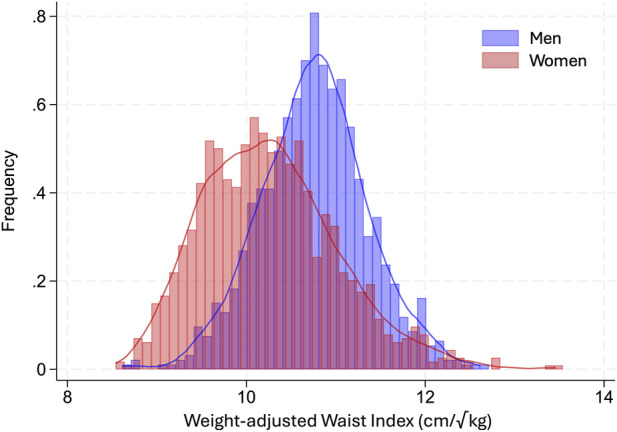
Distribution of Weight-adjusted Waist Index by sex in community-dwelling older adults participating in the Hordaland Health Study (1997–99).

A total of 2,066 participants (55% women), aged 70–72 years, with available cognitive tests scores and anthropometric measurements were included in the analyses. Descriptive characteristics are listed in Table 1. Cognitive test scores and WWI were approximately normally distributed (Supplementary Figure S2). WWI ranged from 8.54 to 13.46 cm/√kg. A clear sex-specific difference in WWI was observed, with higher values in men (Figure 1). Mean (SD) WWI was 10.8 (0.6) cm/√kg in men and 10.3 (0.8) cm/√kg in women.

**TABLE 1:** Descriptive characteristics of the study population (HUSK 1997–99; born 1925–27).

Variables	Total cohort (n = 2,066)	​	Missing (n)
Demographics			
Age, years, median (IQR)	71 (70, 72)	​	0
Sex, n (%)	​	​	0
Women	1,138 (55.0)	​	​
Men	928 (45.0)	​	​
Education, n (%)	​	​	166
Low (≤10 years, primary education)	738 (38.7)	​	​
Medium (11–13 years, secondary education)	806 (42.4)	​	​
High (≥14 years, college or university education)	358 (18.8)	​	​
Cognitive test scores			
KOLT score, mean (SD), range	35.3 (8.2)	2–65	0
COWAT score, mean (SD), range	15.1 (5.5)	1–39	0
m-DST score, mean (SD), range	10.3 (4.2)	1–24	0
Anthropometrics and body composition			
Weight-adjusted Waist Index, WC/√kg, mean (SD), range	10.5 (0.7)	8.5–13.5	0
WC, cm, mean (SD), range	89.5 (12.0)	54.0–138.0	0
WHR, mean (SD), range	0.9 (0.1)	0.6–1.2	0
BMI, kg/m^2^, mean (SD), range	26.1 (3.9)	14.2–44.6	0
BF%, mean (SD), range	34.2 (10.3)	5.8–61.6	634
Lean mass index, kg/m^2^, mean (SD), range	16.2 (2.3)	9.9–23.5	634
Weight, kg, mean (SD), range	73.1 (12.9)	34.5–128.0	0
Height, cm, mean (SD), range	167.2 (9.3)	136.0–199.0	0
Lifestyle factors			
Current smoking^a^, n (%)	350 (16.9)	​	12
Physical activity			
Light physical activity, n (%)	​	​	150
None	111 (5.4)	​	​
<1 h/week	144 (7.0)	​	​
1–2 h/week	568 (27.5)	​	​
≥3 h/week	1,093 (52.9)	​	​
Vigorous physical activity, n (%)	​	​	198
None	1,053 (51.0)	​	​
<1 h/week	291 (14.1)	​	​
1–2 h/week	312 (15.1)	​	​
≥3 h/week	212 (10.6)	​	​
General health conditions			
Diabetes, n (%)	132 (6.4)	​	30
Stroke, n (%)	98 (4.7)	​	39
Myocardial infarction, n (%)	211 (10.2)	​	29
Depression, n (%)	37 (1.8)	​	287
Metabolite levels			
CRP, ug/L, median (IQR), range	2.1 (1.1, 4.4)	0.0–69.3	0
Kynurenine-to-tryptophan ratio, median (IQR), range	2.5 (2.2, 2.9)	1.2–7.5	32

^a^ Defined as plasma cotinine level >85 nmol/L. range represents the minimum and maximum value observed in the cohort.

Abbreviations: BMI, body mass index; COWAT, controlled oral word association test; CRP, C-Reactive protein; HUSK, the Hordaland Health Study; IQR, interquartile range; KOLT, kendrick object learning test; m-DST, modified Digit Symbol Test; SD, standard deviation; WC, waist circumference; WHR, waist-to-hip ratio.

### 3.2 Associations between WWI and cognitive test scores

Higher WWI was inversely associated with cognitive test scores across COWAT, KOLT, and m-DST (Table 2). The inverse associations were observed in all regression models, and stepwise adjustment did not materially change the magnitude or direction of the estimates. In the fully adjusted models, a one SD increase in WWI was associated with 0.06 SD lower scores on COWAT and KOLT, and 0.09 SD lower score on m-DST. When applying absolute values for WWI and cognitive test scores, each one-unit increase in WWI was associated with 0.5 lower points on COWAT, 0.7 lower points on KOLT, and 0.5 lower points on m-DST (Supplementary Table S2). Across the observed range of WWI in the study population (approximately 5 cm/√kg), this corresponded to estimated differences of 2.5 points for COWAT, 3.5 points for KOLT, and 2.5 points for m-DST.

**TABLE 2:** Anthropometric and body composition measures and cognitive test scores.

Variables	Model 1	Model 2	Model 3
	ß (95% CI)	ß (95% CI)	ß (95% CI)
COWAT (verbal fluency)			
WWI	**−0.08 (−0.13, −0.04)**	**−0.06 (−0.11, −0.02)**	**−0.06 (−0.11, −0.02)**
WC	−0.06 (−0.10, 0.01)	−0.04 (−0.09, 0.01)	−0.04 (−0.09, 0.01)
WHR	**−0.08 (−0.14, −0.03)**	**−0.07 (−0.12, −0.01)**	−0.05 (−0.11, 0.01)
BMI	−0.04 (−0.08, 0.01)	−0.03 (−0.07, 0.02)	−0.02 (−0.07, 0.02)
BF%	−0.12 (−0.47, 0.23)	0.00 (−0.04, 0.04)	0.00 (−0.03, 0.04)
LMI	−0.13 (−0.33, 0.06)	−0.15 (−0.35, 0.04)	−0.16 (−0.35, 0.04)
KOLT (memory)			
WWI	**−0.08 (−0.13, −0.04)**	**−0.06 (−0.11, −0.02)**	**−0.06 (−0.11, −0.02)**
WC	−0.05 (−0.09, 0.003)	−0.02 (−0.07, 0.03)	−0.02 (−0.07, 0.03)
WHR	**−0.08 (-0.14, −0.02)**	−0.05 (−0.11, 0.003)	−0.05 (−0.11, 0.01)
BMI	−0.01 (−0.05, 0.03)	0.01 (−0.04, 0.05)	0.01 (−0.04, 0.05)
BF%	−0.01 (−0.07, 0.04)	0.01 (−0.04, 0.06)	0.01 (−0.04, 0.07)
LMI	0.09 (−0.20, 0.38)	0.07 (−0.22, 0.36)	0.08 (−0.22, 0.37)
m-DST (processing speed)			
WWI	**−0.10 (−0.14, −0.05)**	**−0.09 (−0.13, −0.04)**	**−0.09 (−0.12, −0.04)**
WC	−0.03 (−0.08, 0.02)	−0.02 (−0.07, 0.02)	−0.02 (−0.07, 0.03)
WHR	**−0.08 (−0.13, −0.03)**	**−0.07 (−0.12, −0.01)**	**−0.07 (−0.13, −0.01)**
BMI	−0.01 (−0.05, 0.03)	−0.01 (−0.04, 0.03)	−0.01 (−0.05, 0.03)
BF%	0.00 (−0.02, 0.03)	0.01 (−0.02, 0.03)	0.01 (−0.02, 0.04)
LMI	−0.07 (−0.21, 0.08)	−0.09 (−0.24, 0.05)	−0.10 (−0.24, 0.05)

Z-standardized beta-coefficients (ß) and 95% confidence intervals (CI) are from multivariate linear regression analyses. Missing values are imputed by multiple imputations by chained Equation (20 imputations). Number of participants from the Hordaland Health Study included in the analyses are 2,066 for WWI, WC, WHR, and BMI, and 1,432 for BF% and LMI. Bold values indicate statistically significant effect sizes (p-value <0.05).

Model 1: adjusted for sex, age, and education.

Model 2: adjusted for sex, age, education, physical activity level, and current smoking status.

Model 3: adjusted for sex, age, education, physical activity level, current smoking status, myocardial infarction, stroke, diabetes, and depression.

Abbreviations: BF%, body fat percentage; BMI, body mass index; COWAT, controlled oral word association test; KOLT, kendrick object learning test; LMI, lean mass index; m-DST, modified Digit Symbol Test; WC, waist circumference; WHR, waist-to-hip ratio.

### 3.3 Inflammatory markers and the association between WWI and cognitive performance

In regression models additionally adjusted for inflammatory biomarkers, the inverse association between WWI and cognitive test performance remained largely unchanged (Table 3). In the fully adjusted models, WWI was inversely associated with scores on COWAT, KOLT, and m-DST, with effect estimates similar to those observed prior adjustment for inflammatory markers. KTR was inversely associated with COWAT score, but not with KOLT or m-DST scores. CRP was not associated with performance on any of the cognitive tests.

**TABLE 3:** Weight-adjusted waist index (WWI) and cognitive test scores adjusted for inflammatory markers.

Variables	COWAT (verbal fluency)	KOLT (memory)	m-DST (processing speed)
	ß (95% CI)	ß (95% CI)	ß (95% CI)
WWI	**−0.06 (−0.10, −0.01)**	**−0.06 (−0.11, −0.01)**	**−0.09 (−0.13, −0.05)**
C-reactive protein	0.00 (−0.04, 0.04)	0.01 (−0.04, 0.05)	0.04 (−0.003, 0.08)
KTR	**−0.05 (−0.09, −0.01)**	−0.04 (−0.09, 0.001)	−0.04 (−0.08, 0.001)

Standardized beta-coefficients (ß) and 95% confidence intervals (CI) are from multivariate linear regression analyses. Missing values are imputed by multiple imputations by chained Equation (20 imputations). N = 2066. The regression analyses are adjusted for sex, age, education, physical activity level, current smoking status, myocardial infarction, stroke, diabetes, depression, and inflammatory biomarkers [C-Reactive protein and kynurenine-to-tryptophan ratio (KTR)]. Bold values indicate statistically significant effect sizes (p-value <0.05).

COWAT, controlled oral word association test; KOLT, kendrick object learning test; m-DST, modified Digit Symbol Test; WWI, weight-adjusted waist index.

When CRP and KTR were entered individually into the regression models, the inverse association between WWI and cognitive test scores remained materially unchanged (Supplementary Table S3). KTR was inversely associated with COWAT score only, whereas no associations were observed for CRP across any of the cognitive outcomes.

### 3.4 Associations between body measures and cognitive test scores

BMI, WC, and WHR were normally distributed (Supplementary Figure S3). Table 1 provides descriptive data. WWI was inversely associated with performance across all cognitive tests in fully adjusted models, with the strongest association observed for processing speed (m-DST). In contrast, traditional anthropometric measures, including BMI and WC, were not significantly associated with cognitive test performance (Table 2). WHR showed inverse associations with cognitive scores, reaching statistical significance for m-DST, while associations with COWAT and KOLT were attenuated after full adjustment (Table 2).

Body composition data (BF% and LMI) were available for a subgroup of 1,432 subjects, comprising 748 women and 684 men. The body composition measures were normally distributed (Supplementary Figure S4). Table 1 provides descriptive data. Cognitive test scores were normally distributed within the subgroup with a mean score of 15.2 ± 5.6 points for COWAT, 35.2 ± 8.2 points for KOLT, and 10.4 ± 4.2 points for m-DST, comparable to the scores obtained from the whole cohort. The linear regression analyses revealed no associations between body fat percentage or LMI with cognitive test scores across any of the cognitive domains assessed (Table 2).

## 4 Discussion

In this cross-sectional study of community-dwelling older adults, higher WWI was consistently and inversely associated with cognitive performance across multiple domains including verbal fluency, memory, and processing speed. These associations remained robust after adjustment for sociodemographic factors, lifestyle variables, depression, cardiometabolic conditions, and inflammatory biomarkers.

Our findings are consistent with previous studies reporting inverse associations between WWI and cognitive outcomes. Large cross-sectional and longitudinal studies have demonstrated that higher WWI is associated with poorer cognitive performance, accelerated cognitive decline, and increased risk of cognitive impairment across diverse populations (Li et al., 2024; Qiu et al., 2024; Qian et al., 2025; Lu et al., 2026). In contrast, traditional anthropometric measures including BMI and WC, as well as body composition measures including BF% and LMI, were not associated with cognitive performance in the current study. Although WHR, also reflecting abdominal obesity, was inversely associated with cognitive test scores, these associations were not statistically significant in fully adjusted regression models for COWAT (verbal fluency) and KOLT (memory).

Although most systematic reviews and meta-analyses on obesity and cognition have focused on global cognitive impairment or dementia risk (Phirom et al., 2025) (57), a systematic review of observational studies reported that obesity was most consistently associated with poorer performance in psychomotor performance and speed, visual construction, concept formation, and decision making (Prickett et al., 2015). Available evidence was inconsequent for the cognitive domains reflecting visual memory, verbal memory, and complex attention, and there was no evidence of deficits in domains of general cognitive performance, time judgement, working memory or verbal fluency (Prickett et al., 2015). Evidence from cohort and cross-sectional studies also suggest some variation across cognitive domains. In the Doetinchem Cohort Study, higher BMI and WC were associated with poorer cognitive performance across several cognitive domains, with somewhat larger associations for memory and flexibility compared with processing speed (Te Hoonte et al., 2026). In a large community-based older cohort, Wu et al. found higher central adiposity to associate with accelerated decline in global cognitive function, episodic memory, and processing speed (Wu et al., 2025). Similarly, Qiu et al. reported that higher WWI was associated with poorer performance in cognitive domains reflecting learning, verbal fluency, and processing speed among older adults (Qiu et al., 2024). The strongest and most consistent associations were observed for processing speed and verbal fluency, while associations with delayed recall were less robust after adjustment (Qiu et al., 2024).

Overall, these findings may suggest that obesity is associated with poorer performance across several cognitive domains, however, inconsistencies exist between cohorts which may reflect differences in the adiposity measures applied, cohort characteristics, and cognitive assessments used. Nevertheless, our findings are in line with findings from both Qiu et al. (2024) and Wu et al. (2025), indicating that reduced processing speed is one of the domains most strongly associated with central adiposity. Associations with memory and verbal fluency have also been observed–although with somewhat smaller effect sizes compared to processing speed in our data.

Previous studies examining BMI and WC in relation to cognitive function have reported inconclusive findings (Phirom et al., 2025; Lu et al., 2025), which may partly reflect age-related differences. While midlife obesity has been associated with poorer cognitive outcomes, higher BMI in later life has in some studies been linked to reduced risk of cognitive decline (Wu et al., 2024; Qian et al., 2025), a phenomenon often attributed to reverse causation, whereby weight loss occurs during preclinical phases of dementia (Johnson et al., 2006). Poorer cognitive outcomes have been associated with long-term obesity and weight gain later in life, but also with being underweight or having substantial weight loss in older age (Wu et al., 2024). These findings suggest that the relationship between adiposity and cognition depends on the timing of exposure across the life course.

The observed discrepancies across anthropometric measures may reflect differences in their ability to capture fat distribution. While BMI reflects overall body mass, it does not distinguish between fat and lean mass or account for fat distribution, whereas measures of central adiposity may be more closely linked to metabolic and vascular processes relevant to cognitive function. Previous studies on WC and WHR have yielded inconsistent results (Wu et al., 2024; Qian et al., 2025; Lu et al., 2026), and meta-analyses report conflicting results regarding the association between central obesity and cognitive outcomes (Phirom et al., 2025; Lu et al., 2025). In this context, the consistent association between higher WWI and poorer cognitive performance observed in our study and others (Li et al., 2024; Qiu et al., 2024; Qian et al., 2025; Lu et al., 2026) suggests that this measure may better capture aspects of adverse body composition and metabolic dysfunction compared with traditional anthropometric measures.

Although the underlying mechanisms remain incompletely understood, central obesity–particularly visceral fat–has been linked to several biological pathways associated with cognitive performance, including insulin resistance (Kim and Feldman, 2015), a dysregulated gut microbiota (Loh et al., 2024), vascular dysfunction (Van Der Flie et al., 2018), and chronic low-grade inflammation (Walker et al., 2019). These pathways are also influenced by psychosocial and lifestyle factors, such as psychological stress, depressive symptoms, and socioeconomic conditions, which may also be associated with both adiposity and cognitive function (Livingston et al., 2024). Consistent with these mechanisms, higher WWI has been associated with increased risk of cardiovascular disease (Fang et al., 2023), sarcopenia (Zhou et al., 2024; Lee et al., 2026), and frailty (Cataltepe et al., 2024), which in turn are linked to adverse cognitive outcomes (Vázquez-Lorente et al., 2025; Van Der Flie et al., 2018; Wallace et al., 2020). Furthermore, higher WWI has been associated with elevated levels of neurofilament light chain (Yan et al., 2024), supporting an association between central adiposity and biomarkers of neuronal injury. Taken together, WWI may thus reflect a broader alteration in body composition and systemic metabolic health rather than central adiposity alone. This broader physiological interpretation may partly explain why WWI demonstrated more consistent associations with cognitive performance than conventional adiposity indices in the current study.

Previous analyses from the same cohort demonstrated associations between several inflammatory biomarkers and cognitive performance (Solvang et al., 2019). Based on these findings, we examined whether adjustment for CRP and KTR influenced the observed associations between WWI and cognitive test scores. However, despite the proposed role of inflammation, adjustment for inflammatory biomarkers in the current study did not attenuate the association between WWI and cognitive performance. Although some inflammatory markers were independently associated with specific cognitive test scores, these associations were not consistent across markers or cognitive domains. Together with the stability of the WWI estimates, this suggests that CRP and KTR do not explain the observed associations. As these biomarkers reflect only selected aspects of systemic inflammation and immune activations, primarily the interleukin-6 and interferon-γ-related pathways, the findings do not exclude potential contributors from other inflammatory, metabolic, vascular, or bioenergetic pathways. This may indicate that WWI captures a broader metabolically unfavorable phenotype not fully reflected by the limited inflammatory biomarker panel applied in the present study.

### 4.1 Strengths and limitations

A major strength of the current study is the well-characterized cohort of community-dwelling older adults, with anthropometric measurements conducted by trained healthcare personnel using standardized procedures. Body weight and WC were objectively measured, reducing the risk of misclassification compared to self-reported data. In addition, cognitive function was assessed using a test battery covering multiple cognitive domains.

Compared to previous studies investigating WWI and cognitive function, we also included inflammatory biomarkers. This strengthens the study by enabling exploration of potential mechanistic pathways linking central obesity and cognitive function. The consistency of the associations across stepwise adjusted models supports the robustness of the findings and reduces the likelihood that they are explained by measured confounders alone. Although the standardized regression coefficients were modest in magnitude, indicating small effect sizes, differences in absolute cognitive test scores may still be clinically meaningful at the population level, suggesting that WWI may have a potential as an epidemiological marker of cognitive vulnerability.

Several limitations should be acknowledged. The cross-sectional design cannot establish causality or temporality. Reverse causation cannot be excluded, as early or subclinical cognitive decline may influence body composition, physical activity patterns, or weight, thereby affecting WWI. Furthermore, survival bias may have influenced the findings. Individuals with the most adverse metabolic profiles and highest levels of central adiposity may have been less likely to survive into older age, or participate in the study, and thus being included in the cohort. This could potentially attenuate the observed associations. Although multiple relevant covariates were included, residual confounding cannot be excluded.

Moreover, WWI is an indirect proxy for central adiposity and does not directly quantify visceral fat mass as imaging techniques would. Some degree of exposure misclassification is therefore possible. However, WWI offers a practical and accessible alternative in large-scale epidemiological settings where advanced imaging is not feasible. Furthermore, the study was limited to three cognitive tests reflecting memory, executive function, language, attention, and processing speed. The selection of cognitive tests was based on data availability in the cohort as well as data quality of the tests. However, although limited, these tests cover key cognitive functions relevant for activities of daily life that are essential for independent living beyond basic self-care. As cognition is a broad construct, the findings may not generalize to other cognitive domains such as visuospatial abilities and judgement, or other aspects of executive functions. Exploring these associations in a sample with a more comprehensive neuropsychological test battery would be necessary to elucidate this. Furthermore, a limited panel of inflammatory markers was applied in the study, and thus, our findings do not rule out that other inflammatory markers or inflammatory mechanisms in general may influence cognitive performance. Lastly, the cohort consisted of relatively healthy, community-dwelling older adults with a narrow age range. While this reduces age-related heterogeneity, it limits the ability to explore age-specific effects and may restrict generalizability to younger populations, institutionalized individuals, or populations with greater comorbidity burden.

### 4.2 Clinical and research implications

Population aging and population growth are expected to substantially increase the number of individuals living with mild cognitive impairment and dementia (Nichols et al., 2022). This will impose a considerable burden on affected individuals, their families, and society at large. As no curative treatments for dementia currently exist, prevention remains a central strategy to reduce disease burden. Targeting modifiable risk factors and advancing research into underlying biological mechanisms have been highlighted as key priorities (Nichols et al., 2022). The relationship between weight, obesity, and dementia risk has been difficult to disentangle, and a clearer understanding of which factors and life-course periods that are associated with risk is essential for effective prevention. In this context, our findings suggest that central adiposity, assessed by WWI, may represent a marker of poorer cognitive performance in older adults. Although causality cannot be inferred from this cross-sectional study, these results support the notion that fat distribution–rather than total adiposity–may be more closely associated with cognitive performance. However, as our study cannot exclude reverse causation, longitudinal studies are warranted to clarify the direction of the associations, and whether central obesity is prospectively associated with cognitive performance. Future studies integrating anthropometric measures such as WWI with metabolomic, proteomic, or risk prediction approaches may help clarify the biological mechanisms linking adverse body composition to cognitive decline and dementia-related outcomes.

## 5 Conclusion

Higher WWI, reflecting greater central adiposity, was associated with lower cognitive performance across domains of verbal fluency, memory, and processing speed among Norwegian community-dwelling older adults. Other anthropometric measures were not consistently associated with these cognitive domains. Adjustment for CRP and KTR, markers of low-grade inflammation, did not attenuate the association, suggesting that pathways beyond systemic inflammation may contribute to the observed relationship. In subgroup-analyses, no associations were observed between overall body fat percentage or lean mass index and cognitive performance, further supporting that fat distribution rather than total adiposity may be more closely associated with cognitive health in older adults.

## Data Availability

The data analyzed in this study is subject to the following licenses/restrictions: The data applied in this article are not publicly available due to ethical and privacy restrictions. Access to data from the Hordaland Health Studies (HUSK) may be granted upon reasonable request to the HUSK steering committees and subject to appropriate ethical approvals. Requests to access these datasets should be directed to https://husk-en.w.uib.no/how-to-apply-for-data-access/.
