# Supplementary material for: Weight-adjusted-waist index, inflammation, and cognitive performance in older adults: a cross-sectional analysis from the Hordaland Health Study
Source: Front Aging. 2026 Jul 1;7:1872693. doi: 10.3389/fragi.2026.1872693 (PMC13368758; doi:10.3389/fragi.2026.1872693)
Supplement: Supplementary file 1 [file DataSheet4.docx]

**Supplementary Figure S4.** Distribution of body fat percentage (A) and lean mass index (B) among participants with available dual-energy X-ray absorptiometry scans in the Hordaland Health Study 1997-1999. Distribution of body fat percentage (C) and lean mass index (D) by sex. Kernel density curve shown in C) and D). N = 1432.
