# Supplementary material for: Weight-adjusted-waist index, inflammation, and cognitive performance in older adults: a cross-sectional analysis from the Hordaland Health Study
Source: Front Aging. 2026 Jul 1;7:1872693. doi: 10.3389/fragi.2026.1872693 (PMC13368758; doi:10.3389/fragi.2026.1872693)
Supplement: Supplementary file 2 [file Table1.docx]

**Supplementary Table S1.** Model fit comparison between linear and restricted cubic spline models for associations between body measures and cognitive test scores using AIC and BIC.

| **Body measure** |  | | | **Controlled Oral Word Association Test** | | **Kendrick Object Learning Test** | | **Digit Symbol Test** | |  |
| --- | --- | --- | --- | --- | --- | --- | --- | --- | --- | --- |
|  |  |  |  |  |  |  |  |  |  |  |
| Weight-adjusted waist index | **Model** | **N** | **df** | **AIC** | **BIC** | **AIC** | **BIC** | **AIC** | **BIC** |  |
|  | **Linear** | 2066 | 2 | 12898 | 129010 | 14498 | 14509 | 11786 | 11798 |  |
|  | **RCS 3** | 2066 | 3 | 12899 | 12916 | 14494 | 14511 | 11788 | 11805 |  |
|  | **RCS 4** | 2066 | 4 | 12901 | 12923 | 14494 | 14517 | 11790 | 11813 |  |
|  | **RCS 5** | 2066 | 5 | 12903 | 12931 | 14496 | 14524 | 11792 | 11820 |  |
|  | **RCS 6** | 2066 | 6 | 12903 | 12937 | 14497 | 14531 | 11793 | 11827 |  |
|  | **Test for non-linearity:** | | |  | p = 0.224 |  | **p = 0.015** |  | p = 0.835 |  |
|  | | | | | | | | | |  |
| Waist circumference | **Model** | **N** | **df** | **AIC** | **BIC** | **AIC** | **BIC** | **AIC** | **BIC** |  |
|  | **Linear** | 2066 | 2 | 12915 | 12926 | 14510 | 14522 | 11815 | 11827 |  |
|  | **RCS 3** | 2066 | 3 | 12916 | 12933 | 14512 | 14529 | 11817 | 11834 |  |
|  | **RCS 4** | 2066 | 4 | 12918 | 12941 | 14512 | 14534 | 11819 | 11834 |  |
|  | **RCS 5** | 2066 | 5 | 12920 | 12948 | 14513 | 14542 | 11820 | 11841 |  |
|  | **RCS 6** | 2066 | 6 | 12922 | 12956 | 14515 | 14549 | 11821 | 11855 |  |
|  | **Test for non-linearity:** | | |  | p = 0.534 |  | p = 0.670 |  | p = 0.880 |  |
|  | | | | | | | | | |  |
| Waist-to-hip ratio | **Model** | **N** | **df** | **AIC** | **BIC** | **AIC** | **BIC** | **AIC** | **BIC** |  |
|  | **Linear** | 2066 | 2 | 12913 | 12925 | 14478 | 14490 | 11810 | 11821 |  |
|  | **RCS 3** | 2066 | 3 | 12915 | 12932 | 14480 | 14497 | 11812 | 11829 |  |
|  | **RCS 4** | 2066 | 4 | 12917 | 12939 | 14479 | 14502 | 11813 | 11836 |  |
|  | **RCS 5** | 2066 | 5 | 12917 | 12945 | 14480 | 14508 | 11815 | 11843 |  |
|  | **RCS 6** | 2066 | 6 | 12918 | 12951 | 14482 | 144516 | 11817 | 11851 |  |
|  | **Test for non-linearity:** | | |  | p = 0.482 |  | p = 0.550 |  | p = 0.778 |  |
|  | | | | | | | | | |  |
| Body mass index | **Model** | **N** | **df** | **AIC** | **BIC** | **AIC** | **BIC** | **AIC** | **BIC** |  |
|  | **Linear** | 2066 | 2 | 12917 | 12928 | 14550 | 14561 | 11817 | 11828 |  |
|  | **RCS 3** | 2066 | 3 | 12918 | 12935 | 14551 | 14568 | 11818 | 11834 |  |
|  | **RCS 4** | 2066 | 4 | 12919 | 12942 | 14552 | 14574 | 11819 | 11842 |  |
|  | **RCS 5** | 2066 | 5 | 12921 | 12950 | 14551 | 14579 | 11821 | 11849 |  |
|  | **RCS 6** | 2066 | 6 | 12923 | 12957 | 14551 | 14585 | 11822 | 11855 |  |
|  | **Test for non-linearity:** | | |  | p = 0.340 |  | p = 0.411 |  | p = 0.276 |  |
|  | | | | | | | | | |  |
| Body fat percentage | **Model** | **N** | **df** | **AIC** | **BIC** | **AIC** | **BIC** | **AIC** | **BIC** |  |
|  | **Linear** | 1432 | 2 | 9014 | 9025 | 10055 | 10066 | 8198 | 8209 |  |
|  | **RCS 3** | 1432 | 3 | 9014 | 9030 | 10057 | 10072 | 8199 | 8215 |  |
|  | **RCS 4** | 1432 | 4 | 1016 | 9037 | 10058 | 10079 | 8199 | 8220 |  |
|  | **RCS 5** | 1432 | 5 | 1918 | 9044 | 10058 | 10085 | 8199 | 8226 |  |
|  | **RCS 6** | 1432 | 6 | 1920 | 9051 | 10060 | 10092 | 8200 | 8232 |  |
|  | **Test for non-linearity:** | | |  | p = 0.198 |  | p = 0.412 |  | p = 0.317 |  |
|  | | | | | | | | | |  |
| Lean mass index | **Model** | **N** | **df** | **AIC** | **BIC** | **AIC** | **BIC** | **AIC** | **BIC** |  |
|  | **Linear** | 1432 | 2 | 9017 | 9027 | 10043 | 10054 | 8200 | 8210 |  |
|  | **RCS 3** | 1432 | 3 | 9017 | 9033 | 10045 | 10061 | 8200 | 8216 |  |
|  | **RCS 4** | 1432 | 4 | 9018 | 9039 | 10034 | 10055 | 8199 | 8220 |  |
|  | **RCS 5** | 1432 | 5 | 9019 | 9045 | 10029 | 10056 | 8201 | 8227 |  |
|  | **RCS 6** | 1432 | 6 | 9019 | 9050 | 10032 | 10064 | 8203 | 8234 |  |
|  | **Test for non-linearity:** | | |  | p = 0.246 |  | **p = 0.001^a^** |  | p = 0.206 |  |

All non-linearity tests are conducted with RCS with 3 knots as this non-linear model showed best fit with the data. Lower AIC and BIC values indicate better model fit.

^a^ Test for non-linearity with RSC with 4 knots.

AIC, Akaike Information Criterion; BIC, Bayesian Information Criterion; df, degrees of freedom; N, number of subjects; RCS, Restricted Cubic Splines with 3-6 knots.
