# Supplementary material for: Weight-adjusted-waist index, inflammation, and cognitive performance in older adults: a cross-sectional analysis from the Hordaland Health Study
Source: Front Aging. 2026 Jul 1;7:1872693. doi: 10.3389/fragi.2026.1872693 (PMC13368758; doi:10.3389/fragi.2026.1872693)
Supplement: Supplementary file 3 [file DataSheet3.docx]

**Supplementary Figure S3.** Distribution of **a)** waist circumference, **b)** waist-to-hip ratio, and **c)** body mass index among participants in the Hordaland Health Study 1997-1999. Distribution of **d)** waist circumference, **e)** waist-to-hip ratio, and **f)** body mass index by sex. Kernel density curve shown in d), e), and f). N = 2066.
