# Supplementary material for: Weight-adjusted-waist index, inflammation, and cognitive performance in older adults: a cross-sectional analysis from the Hordaland Health Study
Source: Front Aging. 2026 Jul 1;7:1872693. doi: 10.3389/fragi.2026.1872693 (PMC13368758; doi:10.3389/fragi.2026.1872693)
Supplement: Supplementary file 4 [file Table2.docx]

**Supplementary Table S2.** Associations between weight-adjusted waist index and cognitive test scores among participants in the Hordaland Health Study 1997-1999.

| **Outcome** | **Model 1** | **Model 2** | **Model 3** | **Model 4** |
| --- | --- | --- | --- | --- |
|  | **ß (95% CI)** | **ß (95% CI)** | **ß (95% CI)** | **ß (95% CI)** |
|  |  |  |  |  |
| **COWAT** (verbal fluency) | |  |  |  |
| WWI | -0.61 (-0.94, -0.28) | -0.49 (-0.82, -0.15) | -0.46 (-0.79, -0.12) | -0.44 (-0.79, -0.10) |
|  |  |  |  |  |
| **KOLT** (memory) | |  |  |  |
| WWI | -0.96 (-1.47, -0.46) | -0.72 (-1.23, -0.21) | -0.71 (-1.22, -0.19) | -0.71 (-1.23, -0.19) |
|  |  |  |  |  |
| **m-DST** (processing speed) | |  |  |  |
| WWI | -0.55 (-0.79, -0.30) | -0.50 (-0.75, -0.24) | -0.49 (-0.74, -0.24) | -0.51 (-0.77, -0.26) |
|  |  |  |  |  |

Beta (ß) and 95% confidence intervals (CI) are from multivariate linear regression analyses. Missing values are imputed by multiple imputations by chained equations (MICE, 20 imputations). N = 2066.

Model 1: adjusted for sex, age, and education.

Model 2: adjusted for sex, age, education, physical activity level, and current smoking status.

Model 3: adjusted for sex, age, education, physical activity level, current smoking status, myocardial infarction, stroke, diabetes, and depression.

Model 4: adjusted for sex, age, education, physical activity level, current smoking status, myocardial infarction, stroke, diabetes, depression, and inflammatory biomarkers (C-Reactive protein and kynurenine-to-tryptophan ratio).

COWAT, Controlled Oral Word Association Test; KOLT, Kendrick Object Learning Test; m-DST, modified Digit Symbol Test; WWI, weight-adjusted waist index.
