# Supplementary material for: Weight-adjusted-waist index, inflammation, and cognitive performance in older adults: a cross-sectional analysis from the Hordaland Health Study
Source: Front Aging. 2026 Jul 1;7:1872693. doi: 10.3389/fragi.2026.1872693 (PMC13368758; doi:10.3389/fragi.2026.1872693)
Supplement: Supplementary file 5 [file Table3.docx]

**Supplementary Table S3.** Associations between z-standardised weight-adjusted waist index and cognitive test scores with individual inflammatory markers entered separately into the regression models.

|  | **CRP** | **KTR** |
| --- | --- | --- |
|  | **ß (95% CI)** | **ß (95% CI)** |
|  |  |  |
| **COWAT** (verbal fluency) | |  |
| WWI | **-0.06 (-0.10, -0.01)** | **-0.06 (-0.10, -0.01)** |
| Inflammatory marker | -0.01 (-0.05, 0.03) | **-0.05 (-0.09, 0.01)** |
|  |  |  |
| **KOLT** (memory) | |  |
| WWI | **-0.06 (-0.11, -0.02)** | **-0.06 (-0.11, -0.01)** |
| Inflammatory marker | -0.004 (-0.05, 0.04) | -0.04 (-0.08, 0.001) |
|  |  |  |
| **m-DST** (processing speed) | |  |
| WWI | **-0.09 (-0.14, -0.05)** | **-0.08 (-0.13, -0.04)** |
| Inflammatory marker | -0.03 (-0.01, 0.07) | -0.03 (-0.07, 0.01) |

Standardised beta-coefficients (ß) and 95% confidence intervals (CI) are from multivariate linear regression analyses. Missing values are imputed by multiple imputations by chained equations (20 imputations). N = 2066 participants from the Hordaland Health Study 1997-99. The regression analyses are adjusted for sex, age, education, physical activity level, current smoking status, myocardial infarction, stroke, diabetes, depression, and either C-Reactive protein (CRP) or kynurenine-to-tryptophan ratio (KTR) entered separately. COWAT, Controlled Oral Word Association Test; KOLT, Kendrick Object Learning Test; m-DST, modified Digit Symbol Test; WWI, weight-adjusted waist index.
