# Supplementary material for: Weight-adjusted-waist index, inflammation, and cognitive performance in older adults: a cross-sectional analysis from the Hordaland Health Study
Source: Front Aging. 2026 Jul 1;7:1872693. doi: 10.3389/fragi.2026.1872693 (PMC13368758; doi:10.3389/fragi.2026.1872693)
Supplement: Supplementary file 6 [file DataSheet2.docx]

**Supplementary Figure S2.** Distribution of cognitive test scores and weight-adjusted waist index (WWI) among community-dwelling older adults in the Hordaland Health Study 1997-1999. COWAT, Controlled Oral Word Association Test; KOLT, Kendrick Object Learning Test; m-DST, modified Digit Symbol Test. N = 2066.
