# Supplementary material for: Weight-adjusted-waist index, inflammation, and cognitive performance in older adults: a cross-sectional analysis from the Hordaland Health Study
Source: Front Aging. 2026 Jul 1;7:1872693. doi: 10.3389/fragi.2026.1872693 (PMC13368758; doi:10.3389/fragi.2026.1872693)
Supplement: Supplementary file 7 [file DataSheet1.docx]

**Supplementary Figure 1.** Distritbution of test scores from A) a modified version of the Mini-Mental State Examination( m-MMSE), B) a modified version of the Block Design test (m-BD), and C) the Trail-Making Test Part A (TMT-A) among participants in the Hordaland Health Study 1997-1999.
